# Lung Involvement in Chronic Schistosomiasis with Bladder Squamous Cell Carcinoma

**DOI:** 10.3201/eid2412.180355

**Published:** 2018-12

**Authors:** Anastasia Saade, Edith Carton, Audrey Mansuet-Lupo, Romain Jouffroy, Diane Damotte, Hélène Yera, Marie-Pierre Revel, François Goldwasser

**Affiliations:** Hopital Cochin, Paris, France (A. Saade, E. Carton, A. Mansuet-Lupo, D. Damotte, H. Yera, M.-P. Revel, F. Goldwasser);; Universite Paris Descartes, Paris (A. Saade, R. Jouffroy, M.-P. Revel, F. Goldwasser)

**Keywords:** squamous cell carcinoma, Schistosoma haematobium, bladder, metastasis, chronic disease, lung, biopsy, parasites, parasitic diseases

## Abstract

We report a case of chronic *Schistosoma haematobium* infection with pseudometastatic pulmonary nodules and high-grade squamous cell carcinoma in a 30-year-old man in Mali. Lung biopsies revealed chronic pulmonary involvement of *S. haematobium* and ruled out lung metastases.

A 30-year-old man from Mali, who had immigrated to France a year before, was hospitalized for acute urinary retention. The patient reported isolated hematuria over the preceding month with recent dysuria. He was afebrile and had normal vital signs. Physical examination revealed pelvis tenderness and guarding. The only biologic abnormality was a hypereosinophilia (1,640 cells/mm^3^). Unenhanced computed tomography (CT) revealed linear calcifications on the bladder wall, with a large intraluminal mass infiltrating the left ureter (Figure, panel A). Cystoscopy was typical of schistosomiasis. Anatomopathology revealed urinary schistosomiasis complicated by a high-grade, well-differentiated, keratinized squamous cell carcinoma (SCC) (Figure, panel B). Within the wall, ovoid structures, sometimes calcified, corresponded to bilharzia eggs. We found calcified eggs with a terminal spine resembling *Schistosoma haematobium* in the urine (Figure, panel C). Stool examinations were negative. Schistosomiasis serologic tests were positive in hemagglutination and ELISA and were confirmed by immunoblotting. Chest CT revealed multiple, bilateral, diffuse, infracentimetric, pulmonary, ill-defined nodules (Figure, panels D, E), suggesting metastases. Because secondary lesions contraindicate cystectomy and require aggressive chemotherapy, we performed further investigations.

**Figure:**
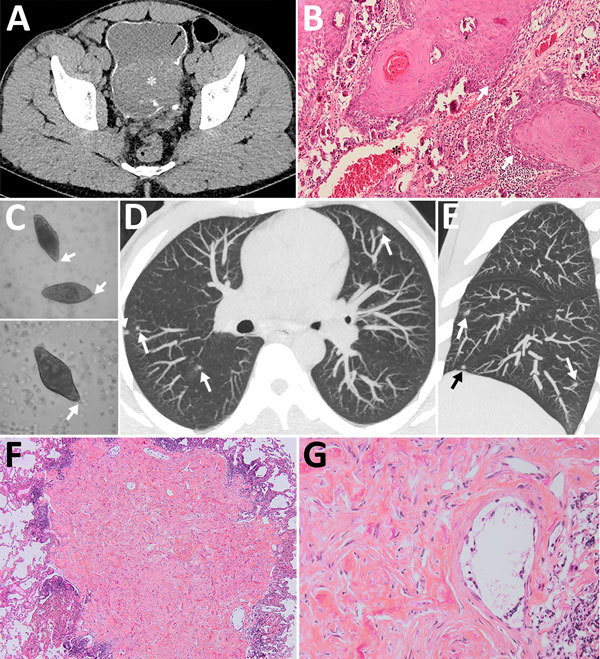
Schistosomiasis-induced squamous cell carcinoma of the bladder with pseudometastatic pulmonary nodules in a 30-year-old man from Mali. A) An unenhanced computed tomography axial image shows diffuse calcification of the bladder wall (arrow). A soft-tissue mass arises from the left posterolateral wall, breaching the calcifications and reaching the perivesical fat (asterisk). B) Anatomopathology slide stained with hematoxylin and eosin (original magnification ×10) of the bladder wall showing massive infiltration by a carcinomatous proliferation of the epidermoid type arranged in islands, or in broad cords in depth (arrows). The cells are large and polygonal, with eosinophilic cytoplasm. The papillomatous malpighian epithelium infiltrates the entire thickness of the chorion and the muscularis into the perivesical fat with venous vascular emboli (asterisk). Within the wall are observed bilharzia eggs, sometimes calcified (small arrow). The chorion is, on the other hand, the seat of a moderate mononuclear inflammatory infiltrate. C) Eggs of *Schistosoma haematobium* in a wet mount of urine concentrate, showing their characteristic terminal spine (original magnification ×400). D, E) Axial (D) and sagittal (E) reconstruction chest computed tomography of the lungs showing blurred ground glass nodules scattered over both lungs. F) A lung subpleural nodule of 0.2 cm greater axis shows at low magnification (original magnification ×4) a fibrous nodule surrounded by lymphoid follicles. G) Hematoxylin and eosin staining of the lung nodule (original magnification ×20) shows inflammatory population, mainly represented by eosinophils and a structure corresponding to a bilharzia egg. No territory suspected of malignancy was visualized.

The patient was HIV-negative. We excluded tuberculosis on the basis of direct examination and culture of sputum and bronchoalveolar lavage fluid. The latter showed 160,000 cells/mL with hypereosinophilia (11%) and alveolar hemorrhage. Ziehl and Grocott stainings were negative. Bacteriologic tests were negative. We detected no tumor cell. We resected a nodule during video-assisted thoracoscopy. Anatomopathology revealed Bilharzia eggs with no carcinomatous lesions (Figure, panels F, G). Another nodule showed similar lesions. The eggshells appeared to be Ziehl-Neelson–negative, typical of *S. haematobium*. Echocardiography ruled out pulmonary hypertension.

We confirmed *S. haematobium* by using molecular analysis of mitochondrial cytochrome oxidase subunit I gene. We excluded *S. haematobium*–*bovis* hybrid species, frequent in Mali, by using internal transcribed spacer region 1 and 2 sequences (*1*).

The patient received 2 courses of praziquantel (40 mg/kg) at 8-week intervals. The tumor was considered free of node involvement and metastases. The patient underwent radical cystectomy with an orthotopic neobladder. Anatomopathology confirmed complete resection of the tumor reaching the muscle layer and the perivesical fat. At 3 months, blood eosinophils were normalized, and chest CT showed complete resolution of all pulmonary nodules. At 6-month follow-up, the patient was still in complete remission.

Schistosomiasis, the second most prevalent endemic parasitic disease, affects ≈230 million persons (*2*). This helminthic infection is caused by a trematode and is also known as snail fever or Bilharzia. The larvae burrow into human skin after contact with contaminated water and migrate within the vascular system to the lung. Through the portal system, mature forms reach venous plexuses surrounding the bladder, and released eggs enter perivesical venules. Adult lifespan averages 3–5 years and can persist up to 40 years.

Acute schistosomiasis occurs 3–6 weeks after infection. Eosinophilia serves as a diagnostic clue. Acute lung lesions are common (*3*). *S. haematobium,* found in Africa and the Middle East, induces urinary schistosomiasis with frequent hematuria. The infection causes fibrosis of the bladder, wall calcification, and hydronephrosis. A major complication is the development of SCC. Schistosomiasis-induced SCC is overrepresented in endemic regions, accounting for only 2.5% of bladder cancer in Western countries (*4**,**5*); it appears as a unique and voluminous mass with low incidence of lymph node involvement and distant metastases at 8%–10% (*4**,**5*). However, the prognosis remains poor, with 90% locoregional recurrence within 3 years. The effective treatment requires radical cystectomy.

Manifestations of chronic disease also involve the lung, and schistosomiasis is the leading cause of pulmonary hypertension, which occurs in 8%–25% of cases (*6**,**7*). A less reported aspect of chronic lung involvement is nodular lesions, which are described in 5% of schistosomiasis cases and are often asymptomatic when isolated (*8*). Belleli was the first to describe *S. haematobium* eggs in the lungs in 1885, stipulating that eggs laying into perivesical plexuses migrate from portal and caval veins to the lungs. Embolized eggs can produce a granulomatous reaction leading to pulmonary fibrosis and obliterative arteriolitis, causing pulmonary hypertension. However, the exact pathophysiology remains unclear and involves different mechanisms. Pulmonary symptoms are more often described with *S. mansoni* and *S.* *japonicum* than *S.* *haematobium*, which preferentially transits through the genitourinary rather than the hepatic portal system (*9*). Radiographic features resemble coins or cavitations, whereas CT shows a ground glass-opacity halo or reticulonodular pattern (*8*). Nodules, considered to be ectopic sites, remain unspecific and indistinguishable from secondary lesions (*6*). The diagnosis of chronic lung involvement is oriented by symptoms of associated end-organ. Treatment with praziquantel is efficient on ectopic lesions.

This case is interesting because of the double presentation of schistosomiasis-induced SCC and chronic lung involvement. Although metastases are less frequent than chronic pulmonary nodules, lung metastases were highly expected in the context of invasive SCC. Each diagnosis involves a different therapeutic approach, and delayed diagnosis can affect the patient’s prognosis. Lung biopsies might be necessary when severe disorders are suspected and avoided when suspicion for other causes, such as tuberculosis and malignancy, is low (*8*). In the latter scenario, PCR on bronchoalveolar lavage fluid might be an alternative in the diagnosis of chronic pulmonary schistosomiasis (*10*).
